# Summary and considerations in genitourinary cancer patient care during the COVID-19 Pandemic

**DOI:** 10.1590/S1677-5538.IBJU.2020.S115

**Published:** 2020-07-27

**Authors:** Francisco Rodríguez-Covarrubias, Ricardo A. Castillejos-Molina, Ana María Autrán-Gómez

**Affiliations:** 1 Instituto Nacional de Ciencias Médicas Nutrición Salvador Zubirán Department of Urology Mexico City Mexico Department of Urology, Instituto Nacional de Ciencias Médicas y Nutrición Salvador Zubirán. Mexico City, Mexico; 2 Hospital Universitario Fundación Jiménez Díaz Department of Urology Madrid Spain Department of Urology, Hospital Universitario Fundación Jiménez Díaz. Madrid, Spain

**Keywords:** Urologic Neoplasms, Therapeutics, COVID-19 [Supplementary Concept], Pandemics

## Abstract

**Purpose:**

To provide a summary and recommendations for the set-up of strategies for cancer patients care in genitourinary oncology clinics during the pandemic and in the recovery period.

**Material and Methods:**

A non-systematic review of available literature on the management of urological malignancies during the COVID-19 pandemic was performed to summarize recommendations to improve the diagnosis and treatment of urological cancers during and after the contingence, including clinical and research aspects.

**Results:**

Urological cancer diagnosis and management should be tailored according to the severity of the COVID-19 crisis in each region and the aggressiveness of each tumor. Clinicians should adhere to strict protocols in order to prioritize the attention of patients with high-risk malignancies while optimizing resources to avoid the saturation of critical care services.

**Conclusions:**

During the COVID-19 pandemic urological cancer care has been severely impaired. For proper patient management, multidisciplinary approach is encouraged tailoring therapy according to COVID-19 regional behavior and local institutional resources. Patients with high-risk malignancies should be prioritized.

## INTRODUCTION

In recent months, the new coronavirus (SARS-CoV-2) disease (COVID-19) became an epidemiological phenomenon that has overwhelmed health care systems globally (1). The negative impact of this pandemic has involved multiple aspects of clinical practice and health services all around the World, including Genito-Urinary Oncology (GUO) clinics.

The consequences of this crisis on cancer patients care is two-fold: a) those diagnosed shortly before or during the outbreak will experience a delay on their treatment; b) new patients with potentially high-risk malignancies will undergo a delayed diagnostic process with the inherent risks due to late approach and therapy, such as progression and losing the chance of curability. In addition, cancer research has been affected, impairing many fundamental steps of clinical investigation, including patient enrolling on clinical trials, follow-up and adverse event assessment (2).

Therefore, our objective is to provide some recommendations for the set-up of strategies for cancer patients care in GUO clinics during the pandemic as well as in the recovery period, considering that some modifications implemented to consultation areas, schedules and facilities might be in place for a while.

## MATERIAL AND METHODS

On May 8^th^, 2020 we performed a non-systematic review of available literature through online search engines (PubMed, Web of Science and Science Direct). We looked at recommendations and the management of urological malignancies during the COVID-19 pandemic. We explored Pubmed database, American Confederation of Urology (CAU) library, European Association of Urology (EAU) recommendations, American Urological Association (AUA) COVID-19 Information Center and American Society of Clinical Oncology (ASCO) Coronavirus resources looking for publications on the aforementioned topics. After analyzing the content of selected peer-reviewed papers, we summarized some recommendations to improve the diagnosis and management of uro-logical cancers during and after the pandemic, taking into account clinical and research issues.

## GENERAL RECOMMENDATIONS

A variety of logistic considerations have to be taken into account during this pandemic when it comes to care of patients with cancer. First, clinicians working in GUO clinics should be aware whether their institutions are becoming temporary centers providing service exclusively to patients with COVID-19 or not. In this case, a strategy to timely refer patients to supporting clinics should be established in order to avoid delay in diagnostic and therapeutic procedures. Second, for those institutions treating both patients with and without COVID-19, clinicians should adhere to strict protocols in order to prioritize the attention of those with high-risk malignancies as well as to optimize resources to avoid the saturation of critical care services.

For the case of cancer care during this emergency, clinical pathways should be tailored according to the severity of the COVID-19 crisis in each region and the aggressiveness of each tumor.

On other hand, the high number of infected health care professionals in Europe suggests that clinicians should wear personal protective equipment properly at all times when performing surgical procedures (3).

### Prostate cancer

#### Diagnosis

According to current estimates, patients at risk of being diagnosed with prostate cancer (PC) are also at risk of having worse outcomes if they get infected with SARS-CoV-2 (i.e. men older than 65 years with comorbidities). Therefore, a reasonable strategy is to delay PC diagnostic process until the critical phase of COVID-19 pandemic has passed (4), especially for those individuals with low-risk features. In contrast, for patients with high-risk characteristics, including rising serum prostate specific antigen (PSA) levels, suspicious digital rectal examination (DRE) or PI-RADS 3 or higher on multiparametric magnetic resonance imaging (mpMRI), an individualized decision--making process is guaranteed (5). If diagnosis is imperative, prostate biopsy should be performed according to each patient's SARS-CoV-2 infection status (5). In non-suspicious cases, prior testing for SARS-CoV-2 is advisable to rule out an asymptomatic infection; if negative, treating physician may proceed with biopsy process, while taking all precautions to avoid transmission during the procedure (due to false negative rates of SARS-CoV-2 testing). On the other hand, patients with suspicious or confirmed COVID-19 should wait until the resolution of infection and testing becomes negative. Regardless of patient's SARS-CoV-2 infection status (including asymptomatic patients), it is strongly advised that health care professionals use complete protection against infection when performing invasive procedures, if possible three-level protection standards (6).

#### Treatment

##### Localized and locally advanced disease

For patients with low-risk disease (T2a, Grade group 1, PSA<10 ng/mL), active surveillance (AS) is the best option. If focal therapy is considered, it may safely be delayed until the pandemic is under control (4).

For intermediate- (T2a-T2c, Grade group 2 or 3, PSA 10-20 ng/mL) and high--risk (T3a, Grade group 4 or 5, PSA >20 ng/mL) patients suitable for radical prostatectomy, a treatment deferment of up to 6 months has not been associated with adverse outcomes (7). If External Radiation Therapy (RT) is considered, the addition of neoadjuvant Androgen Deprivation Therapy (ADT) allows for a safe delay of RT until the resolution of the pandemic while hypofractioned RT may help to reduce health care burden and patient risk of exposure (4).

#### Advanced disease

In case of metastatic hormone-sensitive disease (mHSPC) and first-line therapy for metastatic castration-resistant disease (mCRPC), androgen-receptor-axis targeted therapies (ARATs) in addition to ADT are the preferred option over systemic docetaxel-based chemotherapy to reduce the risk of neutropenia during the pandemic (8). If ARATs have been used previously, a patient-clinician joint decision should be taken to assess the deferment of docetaxel initiation; the individual risk of COVID-19 adverse outcomes; and inherent logistic difficulties regarding the administration of intravenous agents during the pandemic. For patients with mCRPC and bone-only metastases, Radium-223 may be a better alternative if available (8). When possible, the use of glucocorticoids should be minimized (4).

### Urothelial cancer

#### Bladder cancer

##### Diagnosis

Patients with bladder cancer are also at higher risk of adverse outcomes from COVID-19 since they are frequently older than 65 years, commonly former or current smokers and have other comorbidities (i.e. hypertension, obesity). Patients with newly onset macroscopic hematuria should be evaluated with urinary cytology, imaging and office cystoscopy. For individuals with suspected or previously identified low-risk non--muscle invasive bladder cancer (NMIBC), delaying cystoscopic surveillance could be safe (4). In contrast, according to a recent surgical classification designed to improve the management of uro-oncological diseases during the pandemic (3), transurethral resection of bladder tumor (TURBT) should be considered a non-deferable procedure high-risk NMIBC, whether it is diagnostic or therapeutic.

#### Treatment

##### Non-muscle invasive bladder cancer

The evolution of low-grade NMIBC tends to be indolent in the majority of cases. Thus, during the COVID-19 pandemic, AS is an acceptable option (4).

High-grade NMIBC could be better treated with TURBT and induction Bacillus Calmette-Guérin (BCG) intravesical instillations (6 weekly) followed by one maintenance course (3 weekly). Re-staging TURBT (second look) is strongly advised in high-risk patients particularly if muscle was absent in the baseline resection (4). Radical cystectomy (RC) for high-risk NMIBC should be discouraged during the acute phase of the pandemic.

##### Muscle invasive bladder cancer

Delaying RC in muscle invasive bladder cancer (MIBC) for more than 90 days is associated with worse outcomes. Thus, surgical management in this scenario should be individualized. The risk-benefit balance of neoadjuvant chemotherapy should be assessed to reduce toxicity. Bladder-sparing trimodality treatment could be an option for MIBC during the COVID-19 crisis (4).

##### Advanced bladder cancer

Multidisciplinary approach is crucial in this situation in order to avoid treatment interruptions. If platinum-based chemotherapy is recommended, risks and benefits should be evaluated (4). In properly selected patients with low-volume disease, the deferment of chemotherapy could be considered (8).

#### Upper tract urothelial carcinoma

##### Diagnosis

During this pandemic, the workup of patients with suspected upper tract urothelial carcinoma (UTUC) should be limited to urinary cytology and computed tomography (CT) urography (3). Diagnostic ureteroscopy should be performed only if it is mandatory.

##### Treatment

In case of low-risk UTUC, nephron--sparing strategies could be considered (5). For high-risk UTUC, radical nephroureterectomy could be safely delayed for up to 12 weeks in recently diagnosed patients (4). Therefore, treatment options should be discussed carefully in a multidisciplinary team.

### Renal-cell carcinoma

#### Diagnosis

As with PC and BC, renal-cell carcinoma (RCC) patients are at higher risk of COVID-19 due to comorbidities (older age, hypertension). The initial workup, including renal mass biopsy, for patients with small renal masses (<4 cm) could be delayed until the contingency is over (4). For patients with gross hematuria or back pain, an imaging-based approach is advised.

#### Treatment

##### Localized and locally advanced RCC

AS is the preferred alternative for SRM and T1 tumors in the current emergency. For tumors suitable for nephrectomy (T1b and T2), delays of 3 to 6 months appear not to be deleterious (4). Patients with T3 tumors, especially those with inferior vena cava involvement and those at higher risk of symptomatic or oncological progression should be prioritized (3).

##### Metastatic RCC

During the current epidemiological situation, upfront cytoreductive nephrectomy is discouraged and should only be reserved for severely symptomatic patients due to untreatable pain or gross hematuria with clot retention (4). For asymptomatic patients with lowand intermediate-risk International Metastatic RCC Database Consortium (IMDC) disease, AS could be considered. In poor-risk patients, oral vascular endothelial growth factor (VEGF) targeted therapy is associated with lower risk of toxicity-related admissions in comparison to immunotherapy agents (4, 8).

#### Testicular and penile cancer

Information regarding the effect on therapy delay in these two malignancies is lacking. Given their natural history, it seems advisable not to postpone primary surgical management in localized disease. For intermediate and poor prognosis metastatic germ-cell tumors, chemotherapy should be administered without delay.

## FUTURE DIRECTIONS

Among the invaluable devastating effects of this outbreak is the delay of thousands of elective surgeries and other outpatient invasive procedures all around the World in an unprecedent effort to save resources to face the contingency. Although urological practice has been impacted in different aspects (9) some learnings left by this emergency arise: 1) surgical teams are encouraged to adopt comprehensive preoperative medical optimization protocols to reduce the risk of postoperative complications while enhancing the use of hospital resources; 2) diagnostic testing that do not contribute to improve patient outcomes should be eliminated (i.e. daily chest radiographs, daily laboratory tests, urinary cultures to assess asymptomatic bacteriuria or isolated fever in a recently operated patient); 3) the establishment of formal telemedicine programs will allow for care provision while reducing the need for patient mobilization and exposure to dangerous environments. In summary, beyond the tragic effects of this emergency, several opportunities for improvement have arisen as a result of the crisis (10).

## CANCER RESEARCH DURING COVID-19 PANDEMIC

Basic and clinic research is likely to be severely affected by a pandemic. There will most likely be a decrease in trial initiations and accruals and the pace of progress will slow (11). However, the impact will be magnified, perhaps exponentially by protocol deviations and violations for missed and delayed visits, leading to countless queries and estimated dates of confinement. During the Pandemic, there is a need to carefully reconsider the clinical GUO research processes and procedures that contribute to data integrity and patient safety versus tasks that might ultimate detract from cancer research goals. On March 18 2020, the FDA published guidance for industry, investigators and institutional review boards on conduct of clinical trials of medical products during the COVID-19 Pandemic (12).

## CONCLUSIONS

The COVID-19 pandemic represents one of the biggest challenges to modern health care history. Urological cancer care has been severely impaired. For proper patient management during this pandemic, multidisciplinary approach is encouraged. Treatment should be tailored according to COVID-19 regional behavior and local institutional resources. Patients with high-risk malignancies should be prioritized. This emergency represents a great opportunity to improve daily clinical practice.
